# Commentary on: Necrosis Following Dermal Injection of Lyophilized Exosomes: A Case Report

**DOI:** 10.1111/jocd.70507

**Published:** 2025-10-13

**Authors:** İsmail Hakkı Ünal, Ahmet Tecik, Anıl Çağrı Tuna, Nedim Uysal, Zeyneb Tecik, Aslan Yürekli

**Affiliations:** ^1^ Department of Dermatology University of Health Sciences, Gulhane Training and Research Hospital Ankara Turkey

**Keywords:** aesthetic complications, commentary, dermatology, exosomes, necrosis

Dear Editor,

1

We read with great interest the article titled “Necrosis Following Dermal Injection of Lyophilized Exosomes: A Case Report”, which presents an important and timely report on the complications associated with exosome‐based aesthetic products. The authors are to be commended for highlighting a rare and serious adverse event of ischemic necrosis following intradermal exosome injections. However, we would like to raise several points that may strengthen the discussion and guide future clinical interpretation.

First, the clinical diagnosis of ischemic necrosis, though plausible, is presumptive and unconfirmed by histopathology. Though the patient refused biopsy, this drawback can be emphasized further. The causative mechanism may also encompass immunological or toxic injury and not just vascular compromise. Importantly, local immunologic reactions may occur even in the absence of systemic findings. In the same way, vascular imaging (e.g., Doppler ultrasonography) might have furnished confirmatory evidence of vascular occlusion or compromise [1].

Second, an infection was excluded clinically, but no microbial cultures or molecular investigations were conducted. Inasmuch as the post‐injection necrosis may sometimes be caused by atypical infections, especially by *Mycobacterium* species, broader exclusion would thus have increased the diagnostic certainty [2]. Although atypical infections such as *Mycobacterium* species may not be the most likely explanation given the rapid onset and clinical appearance, appropriate microbiological investigations would still have been warranted to definitively exclude an infection‐related etiology.

Third, the pathophysiologic explanation centers on vascular compromise such as hyaluronic acid filler–induced necrosis. Mechanical or pressure‐related vascular injury, an additional plausible mechanism in the context of multiple dermal punctures, was not considered. Indeed, a Nicolau‐like mechanism, as described with hyaluronic acid injections [3], could also be speculated in this case, where multiple dermal punctures and potential intra‐arterial entry may have contributed to the rapid ischemic necrosis. The exosome products, however, lack an available reversal agent like hyaluronidase, and the formulation (e.g., the cord blood–derived conditioned media) brings into the equation the danger of immunologic reactions [4]. A widened discussion of these mechanisms potentially may have allowed a better nuanced comprehension.

Finally, the management strategy warrants discussion. Given that the authors themselves concluded the case was most consistent with vascular compromise rather than a systemic reaction, potential therapeutic approaches aimed at reversing or mitigating vascular ischemia (such as vasodilators, antiplatelet therapy, or hyperbaric oxygen) could have been discussed [5]. Furthermore, although the role of early laser therapy in scar management has been demonstrated, its use in a lesion of uncertain etiology and as a monotherapy lacks sufficient justification [6].

In sum, although this case importantly highlights the dangers of unregulated exosome injectables, the lack of histological verification, vascular evaluation, and microbiological ruling out decreases the confidence of the diagnostic assignment. This case most strongly underlines the real dangers of unregulated exosome‐based therapies and reinforces the need for strict regulation and systematic safety testing prior to their use in clinical dermatology.

## Author Contributions

All authors contributed to the conception, literature review, drafting, and critical revision of the manuscript. All authors approved the final version of the manuscript.

## Ethics Statement

This correspondence did not involve human or animal research requiring ethical approval. No patient photographs are included.

## Consent

The authors have nothing to report.

## Conflicts of Interest

The authors declare no conflicts of interest.

## Data Availability

Data sharing not applicable to this article as no datasets were generated or analysed during the current study.

## References

[1] M. A. Munia , C. G. Munia , M. B. Parada , J. Ben‐Hurferraz Parente , and N. Wolosker , “Doppler Ultrasound in the Management of Vascular Complications Associated With Hyaluronic Acid Dermal Fillers,” Journal of Clinical and Aesthetic Dermatology 15, no. 2 (2022): 40–43. PMC8884183 35309877

[2] M. G. Houngbédji , M. Boissinot , G. M. Bergeron , and J. Frenette , “Subcutaneous Injection of *Mycobacterium ulcerans* Causes Necrosis, Chronic Inflammatory Response and Fibrosis in Skeletal Muscle,” Microbes and Infection 10, no. 12–13 (2008): 1236–1243, 10.1016/j.micinf.2008.07.041. 18762268

[3] P. Andre and E. Haneke , “Nicolau Syndrome due to Hyaluronic Acid Injections,” Journal of Cosmetic and Laser Therapy 18, no. 4 (2016): 239–244, 10.3109/14764172.2016.1157260. 26963701

[4] R. H. Mahmoud , E. Peterson , E. V. Badiavas , M. Kaminer , and A. E. Eber , “Exosomes: A Comprehensive Review for the Practicing Dermatologist,” Journal of Clinical and Aesthetic Dermatology 18, no. 4 (2025): 33–40. PMC12007658 40256340

[5] G. Murray , C. Convery , L. Walker , and E. Davies , “Guideline for the Management of Hyaluronic Acid Filler‐Induced Vascular Occlusion,” Journal of Clinical and Aesthetic Dermatology 14, no. 5 (2021): E61–E69. PMC8211329 34188752

[6] K. E. Karmisholt , A. Haerskjold , T. Karlsmark , J. Waibel , U. Paasch , and M. Haedersdal , “Early Laser Intervention to Reduce Scar Formation – A Systematic Review,” Journal of the European Academy of Dermatology and Venereology 32, no. 7 (2018): 1099–1110, 10.1111/jdv.14856. 29419914

